# Prescriptions and Dispensing.—Ointments

**Published:** 1908-10-03

**Authors:** 


### Therapeutics.

### PRESCRIPTIONS AND DISPENSING.—Ointments.

When a basis of a fatty or oily nature is employed, the resulting compound, if solid or semi-solid, is an ointment. Although in the case of any external application no question of uniform division into doses can arise, it is not less important that the active ingredient or ingredients should be uniformly distributed throughout the whole; it is also of importance that the medicament should reach the patient unaltered, any undesirable chemical decomposition being guarded against with as much care as in making a mixture for internal use.

Since, however, the basis of an ointment is not nearly so favourable a vehicle for chemical reaction to occur in as is a liquid in which two substances are dissolved, there is much less likelihood of incompatible substances being brought into reaction, and the prescription and dispensing of ointments does not, therefore, present many difficulties due to the occurrence of chemical changes.

The first and invariable requirement when a solid is to be mixed with a fatty basis is that it should be powdered as finely as possible. This is not only with a view to its even distribution, but also to avoid the irritation that might be caused by applying an ointment in which solid particles of an appreciable size were present.

The official Unguentum Gallae, Unguentum Plumbi Acetatis, and several others belong to this class. Such ointments may either be made in a mortar with a pestle, or on a slab of marble or glazed earthenware by working the ingredients together with a spatula. For quite small quantities the latter is perhaps the better method; for amounts exceeding an ounce the mortar and pestle are preferable.

If a substance is not easy to powder finely, and is, on the other hand, readily soluble in an inert liquid, it is best to dissolve it in a small quantity of the latter and then mix the solution with the fat. This is the method followed in the official Unguentum Potassii Iodidi. When it is desired to make an unofficial ointment of one's own prescribing, one must judge from the nature of the substances whether it is better to dissolve them or not. A volatile solvent that might evaporate and leave the substance in crystals must obviously be avoided.

Some solids, such as extracts, cannot be powdered finely; in order to secure thorough mixing and uniformity in such a case the solid extract may be dissolved in or mixed with a small quantity of the same kind of liquid as was originally employed in making the extract. Thus an aqueous extract should be rubbed quite smooth with enough water to make it a rather thick liquid; for an alcoholic extract a little rectified spirit may be used similarly; the liquid so produced may then be worked in with the basis of the ointment. This is practically the method employed in the official Unguentum Belladonnæ and with slight differences in Unguentum Conii and Unguentum Hamamelidis; the liquid extract is evaporated to a small bulk and mixed with the basis, instead of the solid extract being used.

When the active ingredient is a liquid, the preparation of the ointment is usually very simple, and is best performed with a spatula on a slab. The Unguentum Plumbi Subacetatis is an example.

The various bases used for ointments differ greatly in their capacity for taking up water and other liquids to form a homogeneous ointment. *Adeps Lanæ* (wool fat), whose hydrated form, Adeps Lanæ Hydrosus, or hydrous wool fat, is practically the same thing as lanoline, and contains 30 per cent. of water, excels all other bases in this respect. If it is found that a larger quantity of a liquid has been put into an ointment than some other basis will take up, the replacement of part of the lard, soft paraffin, or whatever basis has first been tried by an equal weight of Adeps Lanæ, will make the dispensing easy.

Unguentum Chrysarobini is an example of an ointment in which the medicament is soluble in the basis. In order to obtain complete solution, and also uniformity, it is usually necessary to melt the basis, and a risk then arises that an ingredient may be soluble in the hot molten fat, but may crystallise out more or less completely on cooling. Heating is also inadvisable when an ingredient is either volatile or decomposed at the temperature to which the basis would be raised. In the case of chrysarobin, if a considerably larger proportion of this active principle is used than is contained in the official ointment, it may still be dissolved in the hot benzoated lard, but on cooling part of it will slowly crystallise out. The ointment will then be no longer homogeneous, and the crystals may be very irritating in the cases of psoriasis, and so forth, in which the ointment is likely to be used. When there is any likelihood of such crystallisation occurring it is best to powder the substance very finely, and mix it with the basis without melting; or, if a calculation is made as to how much will remain dissolved when the ointment is cold, the practitioner may dissolve this amount in the basis with the aid of heat, and after the ointment so produced has thoroughly cooled, the remainder of the medicament may be incorporated with it in the state of fine powder.

Glycerin is often a very useful ingredient, both for assisting in rubbing a substance to a smooth powder, for dissolving it, and for preventing crystallisation. It is used for one or more of these purposes in the official Unguentum Acidi Carbolici, Unguentum Iodi, and Unguentum Sulphuris Iodidi, and it is a very suitable solvent for mercuric chloride when this is required in the form of an ointment.

Alkaloids are generally more soluble in a fatty basis than are their salts. If an alkaloidal salt is preferred to the alkaloid itself it is best to dissolve it in a very small quantity of water before working it in with the basis. In the official alkaloidal ointments, those of aconitine, atropine, cocaine, and veratrine, the alkaloid is first dissolved in oleic acid, forming the oleate, and this is then worked in with the lard.

(*To be continued.*)

